# Numerical Simulation and Experimental Research on Temperature Distribution of Fillet Welds

**DOI:** 10.3390/ma13051222

**Published:** 2020-03-09

**Authors:** Shanchao Zuo, Ziran Wang, Decheng Wang, Bing Du, Peng Cheng, Yicheng Yang, Ping Zhang, Ning Lang

**Affiliations:** 1 China Productivity Center for Machinery, China Academy of Machinery Science & Technology, Beijing 100044, China; b20170183@xs.ustb.edu.cn (S.Z.); pengcheng080@163.com (P.C.); zhangping9409@163.com (P.Z.); ninglang@hnu.edu.cn (N.L.); 2 School of Materials Science and Engineering, University of Science and Technology Beijing, Beijing 100083, China; 3 Robot Department, Harbin Welding Institute, Harbin 150028, China; welding_wzr@126.com (Z.W.);

**Keywords:** temperature distribution, fillet welds, double ellipsoidal heat source, sensitivity analysis, regression analysis

## Abstract

In this paper, a matrix equation for the welding heat source model was proposed to calculate the fillet welds temperature distribution based on the penetration depth and molten width. A double ellipsoid heat source model of fillet weld was established firstly by physical experiment and simulation calculation, and then the orthogonal experiment was constructed based on the previous calculation methods and experimentally measured data. Finally, the matrix equation of the heat source model parameters was obtained by regression analysis based on the joint penetration and width. The experimental and numerical simulation of the temperature distribution had been performed for the fillet weld and the results show that (1) the heat flux increases in one direction, while, oppositely, it decreased in another direction; (2) simulation results were highly in accordance with experiments results. The results indicated that the double ellipsoidal heat source model calculated by the matrix equation is quite appropriate for predicting the transient temperature distribution on the fillet welds for the gas metal arc welding process.

## 1. Introduction

With the integration of traditional industries and the Internet, the manufacturing industry is gradually transforming from traditional mass assembly line production to mass customization production, ushering in a new era of industrial production [1]. While continuously exploring new manufacturing methods, traditional manufacturing methods are also being optimized [2,3]. Welding is one of the most reliable, efficient, and practical metal joining processes, widely used in the manufacture of bridges, ships, equipment parts, etc. [4,5]. However, the welding process is a complex physical and chemical process involving arc physics, heat transfer, metallurgy, and mechanics [6,7]. During the welding process, the welding zone is rapidly heated to melt by the welding heat input and then cooled to room temperature under the action of conduction and radiation, etc. Severe local temperature changes are the main cause of welding stress and deformation [8,9,10].

Welding temperature is one of the important determinants of metallurgy, crystallization, phase transition, and stress-strain field of structural parts [11,12,13], which is the main factor affecting welding quality and production efficiency [14,15]. In conclusion, reasonable temperature distribution is critical for calculating residual stress, deformation, and solidification [16,17]. First of all, the premise of obtaining an accurate temperature field was to formulate a heat source model that was consistent with the actual situation. A welding heat source model was established by Rosenthal’s application of Fourier’s law (that is, point, line, and surface heat sources), which could reasonably calculate the transient temperature distribution at a certain distance from the heat source. Rosenthal’s analysis, however, is less accurate for the temperature in or near the fusion and heat-affected zones because the scheme defined that the physical properties of the material did not change with temperature. To overcome most of these limitations, other forms of heat source models have been proposed. It was worth mentioning that the double ellipsoid heat source model was proposed by Goldak, which could well describe the arc welding heat source model [18].

Due to the development of computers, welding numerical simulations has made great progress [19]. A 3-D heat transfer model has been established by Kim et al., in which the temperature, weld pool shape, and the weld pool reinforcement surface during gas–metal arc fillet welding were analyzed. In the establishment of this model, not only the heat transfer from the welding arc was considered, but also the thermal effect of metal droplets was considered by the volume heat source [20]. By summarizing the calculation of the temperature field of the predecessors, the transient temperature analytical equation of the semi-infinite body under the three-dimensional motion heat source was obtained by Fachinotti et al. [21]. Winczek applied the bimodal heat source model to establish a temperature field analytical model, which could well analyze the temperature of the multi-pass GMAW in the infinite body model [22].

Some scholars have proposed some methods for determining the parameters of heat source models based on the geometry of the molten pool. A method named ‘discretely distributed point heat source model’ was developed by Azar to study the relationship between the experimentally measured data and the source model, the model divided the ellipsoid heat source model into the horizontal and vertical directions of the ellipsoidal heat source, and the parameters were determined based on the geometry of the molten pool [23]. Besides, according to the geometry of the oval pool of submerged arc welding, the oval heat source model was proposed by Aniruddha Ghosh, A. et al. [24]. Subsequently, they presented an analytical model for transient temperature distribution of gas metal arc welded plate for the tilted electrode. In that paper, volumetric heat source, heat transfer from the electrode and convective heat losses from the welded plate surface had been considered [25]. 

The total number of welds in a machine tool manufactured by welding is 1027, among which the total number of fillet welds is 905, reaching 89%. Except for ribbed welds, which are contact welds, the rest are working welds. The welding quality of fillet welds is one of the decisive factors for the manufacturing quality of welding machine tools. Due to the complexity of the machine tool’s structure and the service load, the manufacturing of the welded structural machine tools required more numerical simulations and optimization of the welding process. However, previous numerical simulation and experimental efforts to butt welding with a rectangular workpiece or ignored asymmetric welded structures [26,27]. In fillet welding, the complexity of the welding process was often augmented by the complicated joint geometry containing an asymmetric welded pool morphology. During the welding process of fillet welds, heat input, heat transfer, loss (including conduction, radiation, etc.) were asymmetrically distributed [20]. All these factors must be taken seriously in order to accurately calculate the transient temperature field, stress field, and deformation.

In this work, a matrix equation was proposed, which is an equation to calculate the heat source model based on the penetration depth and molten width. The regression analysis was used to study the sensitivity of the parameters of the heat source model. The orthogonal experiment was constructed, which was a combination of previous calculation methods and experimentally measured data. Then, the parameter equations of the heat source model were constructed by combining the penetration depth and the width of the fillet joint to obtain a more practical double-ellipsoid heat source model. Experimental and microstructural studies of fillet joints were also performed.

## 2. Welding Experiment 

### 2.1. Materials and Welding Parameters

**Figure 1:**
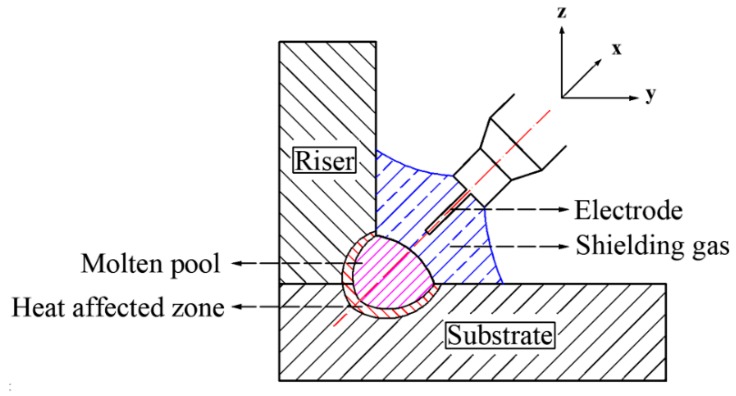
Setup.

The base material was Q235-steel sheet with a thickness of 20 mm and filler wire adopted was ER50-6 with a diameter of 1.2 mm. The composition of workpiece and the filler material was given in Table 1. The welding method was metal active gas arc welding (MAG). To maintain the stability of welding process, welding operation was fully done by the automatic welder (kr16, KUKA, Augsburg, Bavaria, Germany), and the welding power source was Fronius TPS5000 (Fronius, Austria). According to the thickness of the welded steel plate and the size of the filled weld set to 5 mm, the welding parameters shown in Table 2 were set. Figure 1 shows the welding setup.

### 2.2. Experiment Method

**Figure 2:**
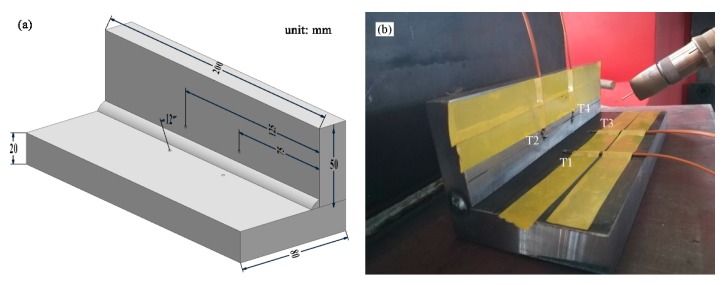
Diagram of the welding and temperature of measuring: (**a**) 3D drawing of the workpiece; (**b**) welding picture.

The temperature on the surface of the workpiece was measured by K-type thermocouples at four points (T_1_, T_2_, T_3_, T_4_), and a Fluke 54-II (Fluke, Everett, WA, USA) multichannel data acquisition module was used for data collection. The data logger was set to record at least one reading per-second from the thermocouples. Figure 2 shows the sample sizes and temperature measurement locations. The longitudinal distance of the temperature measurement points was 50 mm. Meanwhile, it was 75 mm away from the edges of the sample, which prevented the unsteady state from having a large impact on the arc starting and ending. After welding, the fillet welds were cut transversely along the weld. The microstructural images were taken with the scanning electron microscope (Carl Zeiss evo 18, Carl Zeiss, Jena, Germany). 

## 3. Analytical Modeling

Based on ABAQUS software (6.14, Simulia, Providence, RI, USA), a thermal finite element computational procedure is developed to study welding temperature fields during the welding process of fillet-welded joints. The transient heat conduction equation (Equation (1)) is used to analyze the thermal behavior of the MAG process.

(1) $$\frac{\partial }{\partial x}\left(k_{x}\frac{\partial T}{\partial x}\right)+\frac{\partial }{\partial y}\left(k_{y}\frac{\partial T}{\partial y}\right)+\frac{\partial }{\partial z}\left(k_{z}\frac{\partial T}{\partial z}\right)+Q_{t}=\rho c\frac{\partial T}{\partial t}$$

where *k* (assuming *k_x_* = *k_y_* = *k_z_* = *k*) is the thermal conductivity, *Q_t_* is the heat input (caused by the electric arc of the electrode during welding), ρ is the density, and c is the specific heat of the material. It has been assumed that the electrode and the weld plate are of the same material (Q235).

### 3.1. Assumptions and Descriptions of Heat Sources

The welding heat source model is equations used to describe the morphology and heat flux distribution of the welding heat source during the welding process, which will directly affect the temperature distribution. Therefore, the premise of an accurate calculation of temperature field is to establish a model that accurately reflects the actual heat flux distribution.

**Figure 3:**
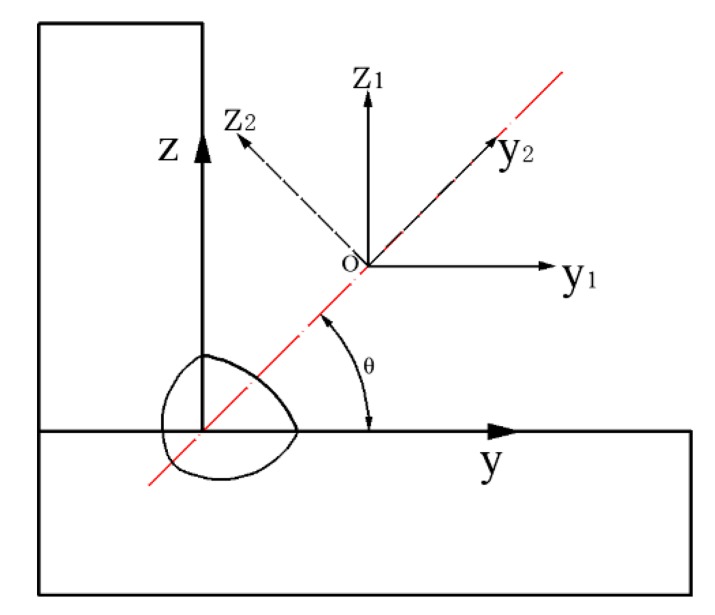
Conversion of coordinates.

The heat source model and its coordinate system are rotated according to the electrode. The derivation is: as presented in Figure 3, the coordinate system *y*_1_*oz*_1_ rotated the *θ* angle anticlockwise around the origin to form the new coordinate system *y*_2_*oz*_2_. For any point, the coordinates in the new coordinate system can be expressed as:

(2) $$\{\begin{array}{c} y_{2}=y_{1}cos\theta +z_{1}sin\theta \\ z_{2}=z_{1}cos\theta -y_{1}sin\theta \end{array}$$

Furthermore, heat flux distribution of fillet welds is based on double ellipsoidal heat source model, it can be described as:

(3) $$\{\begin{array}{c} Q=\eta UI\\ \begin{array}{c} q_{f}=\frac{6\sqrt{3}Qf_{1}}{\pi a_{f}bc\sqrt{\pi }}exp\left[-\frac{3x^{2}}{a_{f}{}^{2}}-\frac{3y_{2}{}^{2}}{b^{2}}-\frac{3z_{2}{}^{2}}{c^{2}}\right]\\ q_{r}=\frac{6\sqrt{3}Qf_{2}}{\pi a_{r}bc\sqrt{\pi }}exp\left[-\frac{3x^{2}}{a_{r}{}^{2}}-\frac{3y_{2}{}^{2}}{b^{2}}-\frac{3z_{2}{}^{2}}{c^{2}}\right] \end{array}\\ f_{1}+f_{2}=2 \end{array}$$

where, *U* is the arc voltage, *I* is the welding current, *η* is the heat source efficiency, *q_f_* is the heat flux density in the front hemisphere, and *q_r_* is the heat flux density in the rear hemisphere, *a_f_* is the length of the front hemisphere of the heat source model, and *a_r_* is the length of the rear hemisphere, *b* is the depth of the heat source model, *c* is the width of the heat source model, *f*_1_ and *f*_2_ of the heat deposited in the front and rear quadrants are needed.

### 3.2. Boundary Conditions

During the welding process, heat is dissipated into the surrounding through convection and radiation from the surface of the workpieces. The heat loss due to convection and radiation over these surfaces is given by:

(4) $$-k(T)\;\frac{\partial T}{\partial n}\;=\;h\;(Tw_{\;}-\;T_{a})\;+\;\varepsilon \sigma \;({T_{w}}^{4}-\;{T_{a}}^{4})$$

where *ε* is the surface emissivity of the material (0.85); *σ* is the Stefan Boltzmann constant (*σ* = 5.67 × 10^−8^ W/(m^2^·K^4^)), *h* is the heat transfer coefficient, *T_w_* is the workpiece temperature (K), *Ta* is the ambient temperature (293 K).

## 4. Results and Discussion

### 4.1. Determine the Heat Source Model Parameters

During the welding process, the magnitude of the heat flux density cannot directly reflect the width and the penetration because of the effect of heat conduction. Since the double ellipsoid model is the basis and main part of the heat source model, it must be studied. 

The double ellipsoid heat source model equation is an exponential equation, by analyzing the response to the heat flux coordinate arguments, the parameter change trend can be obtained. Source model, X, Y, Z three directions of the source parameters *a_f_*, *a_r_*, *b*, *c* is symmetrical. For simplicity, we will only discuss the influence double ellipsoid model for parameter *c*.

(5) $$q=\frac{q_{e}}{a_{f}bc}exp\left[-\frac{3x^{2}}{a_{f}{}^{2}}-\frac{3y^{2}}{{\left(b\right)}^{2}}-\frac{3z^{2}}{{\left(c\right)}^{2}}\right]$$

where, $q_{e}=\frac{6\sqrt{3}Q}{\pi c\sqrt{\pi }}$, by derivation of the equation, it is transformed into the following formula:

(6) $$\frac{\partial q}{\partial c}=\frac{\left(6z^{2}-c^{2}\right)}{a_{f}bc^{4}}exp\left[-\frac{3x^{2}}{a_{f}{}^{2}}-\frac{3y^{2}}{{\left(b\right)}^{2}}-\frac{3z^{2}}{{\left(c\right)}^{2}}\right]$$

**Figure 4:**
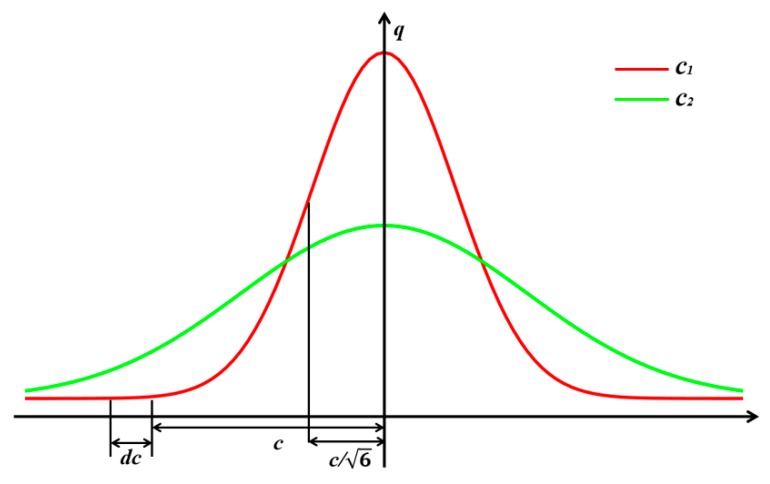
Effect of heat source parameter changes on heat flux density.

Extreme value occurs when $\;c=\sqrt{6}z$. Figure 4 shows the influence of the heat source model parameter *c* on heat flux, where *c*_1_ < *c*_2_. It illustrates that in the range of |*z*| <$\;\frac{1}{\sqrt{6}}c$, as the value of *c* increases, the heat flux density decreases. Conversely, in the range of |*z*| > $\frac{1}{\sqrt{6}}c$, as the value of *c* increases, the heat flux increases in density.

Further, when *z* = 0, $\frac{\partial q}{\partial c}$ constant is negative, it means that when *c* is increased, heat flux at various points obtained, XOY surfaces is reduced. When the width parameter c is increased, the heat flux density in the length direction and depth direction of the molten pool decreases accordingly. The changing trend is similar to other parameters. Thus, it can be concluded that the heat increases the model parameters in an arbitrary direction; the other direction of the heat flux density will decrease.

The melting point of Q235 steel is 1500 °C, so the area above 1500 °C is defined as the liquid region during the simulation. For the heat source model, the input parameters are *a_f_, a_r_, b, c*, and the molten width (*w*) and the penetration depth (*p*), and the regression equation is described as:

(7) $$\{\begin{array}{c} w(a_{f},a_{r},b,c)=x_{1w}a_{f}^{x_{2w}}a_{r}^{x_{3w}}b^{x_{4w}}c^{x_{5w}}\\ p(a_{f},a_{r},b,c)=x_{1p}a_{f}^{x_{2p}}a_{r}^{x_{3p}}b^{x_{4p}}c^{x_{5p}} \end{array}$$

where *x_iw_*, *x_ip_* (*i* = 1, 2,……5) is the undetermined coefficient.

To facilitate regression analysis, the natural logarithm at both ends of the equation are solved. The equation is converted to:

(8) $$\{\begin{array}{c} \ln\left[w\left(\ln a_{f},{\mathrm{lna}}_{r},\ln b,\ln c\right)\right]=\ln x_{1w}+x_{2w}\ln a_{f}+x_{3w}\ln a_{r}+x_{4w}\ln b+x_{5w}\ln c\\ \ln\left[\left(p(\ln a_{f},\ln a_{r},\ln b,\ln c\right)\right]=\ln x_{1p}+x_{2p}\ln a_{f}+x_{3p}\ln a_{r}+x_{4p}\ln b+x_{5p}\ln c \end{array}$$

The orthogonal experiment is constructed, which is a combination of previous calculation methods and experimentally measured data. Data in Table 3 are substituted into the linear equation fitting, there will be:

(9) $$\{\begin{array}{c} w=\frac{18.7031c^{0.1009}}{a_{f}^{0.05674}a_{r}^{0.2320}b^{0.1100}}\\ p=\frac{22.51}{a_{f}^{0.1709}a_{r}^{0.4306}b^{0.0405}c^{0.02857}} \end{array}$$

**Figure 5:**
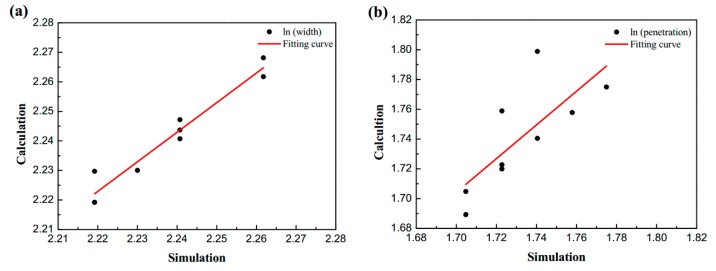
Verification of regression results in sensitivity analysis: (**a**) ln (width); (**b**) ln (penetration).

Figure 5 shows the error verification of the sensitivity regression analysis. The ordinate is the weld formation result calculated by the regression equation, the slopes of the fitting curves in ln (width) and ln (penetration) are close to 45°, which indicates that the calculation results of the regression equation have high accuracy.

For prediction source parameters, *w*, *p*, is a known parameter obtained by the experiment, *a_f_*, *a_r_*, *b*, *c* are parameters to be determined. However, only two equations, the number of arguments must be controlled within the two. Convert it into a matrix form, which can be written as:

(10) $$\left(\begin{array}{c} \ln w\\ \ln p \end{array}\right)=\left(\begin{array}{cc} \begin{array}{ccc} \ln x_{1w} & x_{2w} & x_{3w} \end{array} & \begin{array}{cc} x_{4w} & x_{5w} \end{array}\\ \begin{array}{ccc} \ln x_{1p} & x_{2p} & x_{3p} \end{array} & \begin{array}{cc} x_{4p} & x_{5p} \end{array} \end{array}\right){\left(\begin{array}{cc} \begin{array}{ccc} 1 & \ln a_{f} & \ln a_{r} \end{array} & \begin{array}{cc} \ln b & \ln c \end{array} \end{array}\right)}^{T}$$

In the heat source model parameters, the relationship between *a_f_, a_r_*, and *c* is:

(11) $$\{\begin{array}{c} a_{f}=c\\ a_{r}=3c \end{array}$$

Subsequently, by evaluating and simplifying, Equation can be rewritten as

(12) $$\left(\begin{array}{c} \ln w-\ln x_{1w}-x_{3w}\ln2\\ \ln p-\ln x_{1p}-x_{3p}\ln2 \end{array}\right)=\left(\begin{array}{cc} x_{4w} & x_{2w}+x_{3w}\;+\;x_{5w}\\ x_{4p} & x_{2p}+x_{3p}+x_{5p} \end{array}\right){\left(\begin{array}{cc} \ln b & \ln c \end{array}\right)}^{T}$$

Hence, the matrix equations of the heat source model parameters b and c can be expressed as:

(13) $${\left(\begin{array}{cc} \ln b & \ln c \end{array}\right)}^{T}={\left(\begin{array}{cc} x_{4w} & x_{2w}+x_{3w}\;+\;x_{5w}\\ x_{4p} & x_{2p}+x_{3p}\;+x_{5p} \end{array}\right)}^{-1}\left(\begin{array}{c} \ln w-\ln x_{1w}-x_{3w}\ln3\\ \ln p-\ln x_{1p}-x_{3p}\ln3 \end{array}\right)$$

So,

(14) $${\left(\begin{array}{cc} \ln b & \ln c \end{array}\right)}^{T}={\left(\begin{array}{cc} -0.1100 & -0.1878\\ -0.004051 & -0.6301 \end{array}\right)}^{-1}\left(\begin{array}{c} \ln w-2.9855\\ \ln p-2.6410 \end{array}\right)$$

(15) $${\left(\begin{array}{cc} \ln b & \ln c \end{array}\right)}^{T}=\left(\begin{array}{cc} -9.1918 & 2.7396\\ 0.05910 & -1.6047 \end{array}\right)\left(\begin{array}{c} \ln w-2.9855\\ \ln p-2.6410 \end{array}\right)$$

### 4.2. Model Validation

**Figure 6:**
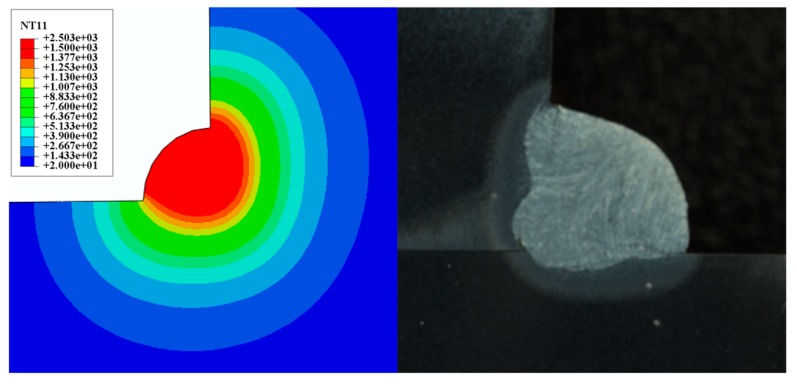
Comparison among calculated results and experimental results (Sample 1#).

**Figure 7:**
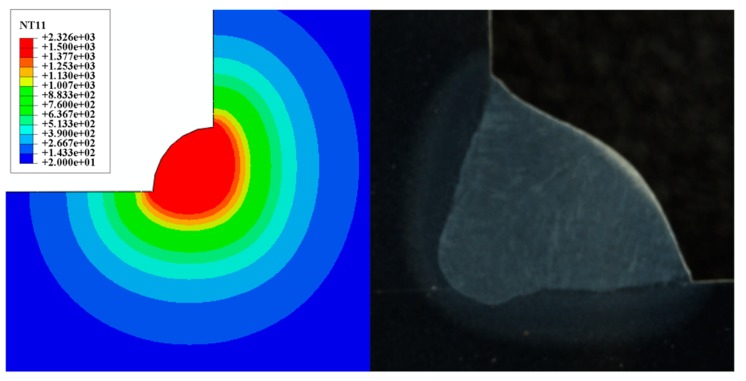
Comparison among calculated results and experimental results (Sample 2#).

**Figure 8:**
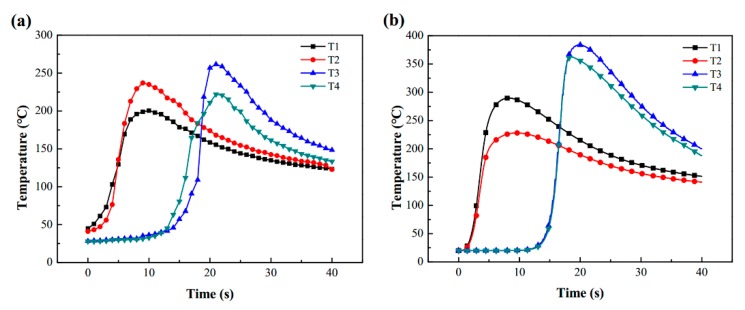
Thermal cycle diagrams for sample 1#: (**a**) measurement temperature; (**b**) simulation temperature.

**Figure 9:**
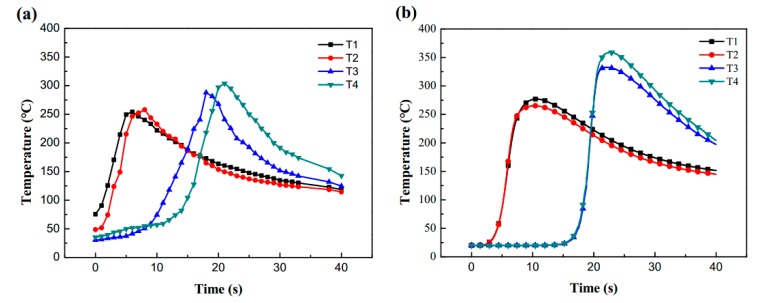
Thermal cycle diagrams for sample 2#: (**a**) measurement temperature; (**b**) simulation temperature.

Equation (15) is applied to solve the heat source model parameters of sample 1# and 2#, and the parameters shown in Table 4 were obtained. Figure 6 and Figure 7 is the fusion line diagram of the simulated and experiment. Sample 1# and 2# both have high fusion line morphology matching. Meanwhile, the thermal cycle diagrams of Figure 8 and Figure 9 are constructed based on the temperature history of temperature measurement points. The comparison between the simulation and experiment is carried out, which shows good agreement, validating the reasonable of the current model. In conclusion, the parameters of the double ellipsoidal heat source model can be accurately calculated by the matrix equation, and the reason of the temperature distribution calculation of the fillet weld will be greatly improved.

Previously, the heat source parameters were surmised by experiment results. That means that the process of solving the parameters of the heat source model is a trial and error process, which consumes a lot of time and energy. However, a matrix equation is proposed to obtain the model parameters of heat source according to the penetrate and width, which will greatly improve the efficiency of determining the parameters of the fillet weld heat source model.

## 5. Conclusions

A matrix equation was proposed for calculating the heat source model based on the weld penetration and width. Compared with the experimental results, the simulation results are highly matched with them. From this paper, the following conclusions can be drawn:

- Sensitivity analysis of double ellipsoid heat source model parameters indicated that the model parameters affect each other. When the heat flux increases in a certain direction, the heat flux will reduce in the other direction.
- Orthogonal experiments constructed with heat source model parameters, penetration depth, and width are calculated using regression analysis, and a matrix formula was obtained. A comparison of the measured and calculated temperature data suggests that consideration of ellipsoid heat source shape for predicting the transient temperature distribution on the welded plate is quite appropriate for the fillet welds process.

## Figures and Tables

**Table 1:** Chemical composition (in weight %).

Chemical Composition	C	Si	Mn	P	S	Cr	Fe
Workpiece (Q235)	0.16	0.14	0.70	0.03	0.02	0.02	Balance
Electrode (ER50-6)	0.08	0.87	1.45	0.012	0.013	0.03	Balance

**Table 2:** Welding parameters.

MAG Setup	Fully Automatic
Shielding gas	80% Ar + 20% CO_2_
Power source	Constant current
Electrode	ER50-6(φ 1.2)
Polarity	DC+
Welding current	170 A
Arc voltage	22 V
Flow rate of shielding gas	15 L/min
Welding speed	300 mm/min
Ambient temperature	20 °C
CTWD	12 mm
Torch angle	45°

**Table 3:** The weld pool size results of heat source parameters sensitivity analysis.

Sample	*a_f_* (mm)	*a_r_* (mm)	*b* (mm)	*c* (mm)	*w* (mm)	*p* (mm)
1#	4	11	4.5	4	9.6	5.7
2#	4	12	5	4.3	9.4	5.6
3#	4	13	5.5	4.5	9.2	5.6
4#	4.5	11	5	4.5	9.6	5.9
5#	4.5	12	5.5	4	9.2	5.7
6#	4.5	13	4.5	4.3	9.3	5.5
7#	5	11	5.5	4.3	9.4	5.8
8#	5	12	4.5	4.5	9.4	5.6
9#	5	13	4	4	9.2	5.5

**Table 4:** Heat source model parameters and simulation results.

-	Parameters (mm)				Penetrate (mm)		Width (mm)	
	*a_f_*	*a_r_*	*b*	*c*	Simulation	Experiment	Simulation	Experiment
1#	4.38	13.14	4.12	4.38	5.3	5.5	9.6	9.4
2#	4.65	13.95	3.07	4.65	5.1	5.3	9.7	9.6
